# The 2019 Ming K. Jeang awards for excellence in *Cell & Bioscience*

**DOI:** 10.1186/s13578-020-00461-9

**Published:** 2020-08-20

**Authors:** Yun-Fai Chris Lau

**Affiliations:** 1 Division of Cell and Developmental Genetics, Department of Medicine, San Francisco VA Health Care System, University of California, San Francisco, 4150 Clement Street, San Francisco, CA 94121 USA; 2 Institute for Human Genetics, University of California, San Francisco, San Francisco, USA

## Abstract

Two articles published by the research groups led by You-Shuo Liu of the Central South University, Changsha, Hunan, and Min Fang of the Huazhong University of Science and Technology, Wuhan, China have been selected as the recipients of the 2019 Ming K. Jeang Award for Excellence in Cell & Bioscience.

We are delighted to announce the 2019 recipients of the Ming K. Jeang Award for Excellence in Cell & Bioscience. Two research groups, who each published an outstanding research article in *Cell & Bioscience* in 2019 [1, 2], have been selected to receive this prestigious award by a panel of Editors, chaired by Dr. Dong Yan Jin. The Ming K. Jeang Awards for Excellence in *Cell & Bioscience* was established in 2011 with a generous endowment from the Ming K. Jeang Foundation to honor outstanding research articles published in *Cell & Bioscience*, the society journal of the Society for Chinese Bioscientists in America (SCBA), a non-profit scientific society, based in North America, https://scbasociety.org/. The selected articles are listed as below.

1. Exosomes from hyperglycemia-stimulated vascular endothelial cells contain versican that regulate calcification/senescence in vascular smooth muscle cells. Shuang Li, Jun-Kun Zhan, Yan-Jiao Wang, Xiao Lin, Jia-Yu Zhong, Yi Wang, Pan Tan, Jie-Yu He, Xing-Jun Cui, Yi-Yin Chen, Wu Huang and You-Shuo Liu. *Cell & Bioscience* 2019 9:1
2. IL-33 ameliorates experimental colitis involving regulation of autophagy of macrophages in mice. Zhongyan Wang, Lifeng Shi, Shuyao Hua, Chang Qi and Min Fang. *Cell & Bioscience* 2019 9:10.

Congratulations to these two groups of investigators for publishing their outstanding research results in *Cell & Bioscience* and winning the 2019 Ming K. Jeang Award.

We are looking forwards to receiving contributions of outstanding research articles and reviews from the scientific community in 2020 and beyond.
